# *Salmonella* Appendicitis in Renal Transplantation

**DOI:** 10.1155/2013/402735

**Published:** 2013-09-04

**Authors:** B. Malone, S. Kleyman, A. Sanni, N. Sumrani, D. Distant

**Affiliations:** Department of Transplantation, SUNY Downstate Medical Centre, Brooklyn, NY 11203, USA

## Abstract

While appendicitis remains one of the commonest surgical diseases, there are relatively few reports following renal transplantation. A 33-year-old man was admitted with diarrhea, fever, and epigastric pain 7 years following a cadaveric renal transplant. CT scanning confirmed a diagnosis of appendicitis which was removed within 24 hours of admission. Histology and blood cultures following surgery confirmed *Salmonella* type b appendicitis. Patient was safely discharged home 5 days following hospital admission.

## 1. Introduction

Acute appendicitis remains one of the commonest causes of an acute abdomen with an estimated 7% of the population developing it in their lifetime [1]. *Salmonella* is rarely associated with appendicitis but can cause it by direct invasion of the appendix, causing inflammation of the appendix, ileum, or lymph nodes [2]. However, appendicitis is very rare following renal transplantation because immunosuppression with corticosteroids is expected to prevent lymphoid hyperplasia [3]. We present a case of *Salmonella* appendicitis in a renal allograft recipient.

## 2. Case Report

A 33-year-old man received a renal allograft from a cadaveric donor on February 5, 1999. The kidney was placed in the right iliac fossa. Maintenance immunosuppression was tacrolimus 4 mg twice daily and prednisone 10 mg once daily.

 On August 10, 2006, he presented with a 1-month history of diarrhea and a 6-day history of fever, epigastric pain, and vomiting. The pain was a burning sensation across the LUQ radiating to the back with associated bile-stained vomitus.

 On examination, he appeared dehydrated with stable vital signs apart from pyrexia of 104.2 F. Abdominal examination elicited tenderness over the RLQ area with normoactive bowel sounds and no flank tenderness. His abdomen was soft with associated guarding and rebound.

Laboratory tests showed a normal white count (8,500/mm^3^) with a mild left shift in the neutrophil bands and a slight increase in his creatinine (2 mg/dL) from baseline.

 The CT scan suggested appendicitis, and patient was scheduled for a laparoscopic appendectomy with possible conversion into an open procedure.

 On open laparotomy, we found a grossly inflamed appendix with no signs of perforation or abscess formation. Postoperatively, histology of the specimen and blood cultures confirmed *Salmonella* group b bacteria infection. This was successfully treated with antibiotics, and patient was discharged home 5 days later.

## 3. Discussion

Following renal transplantation, gastrointestinal complication is the second most common event after infection [4]. Significant mortality as high as 60% has been described in the literature [5]. 

 The estimated lifetime risk of developing appendicitis in the general population is about 8.6% for males and 6.7% for females [6]. Its etiology has been proposed to be due to hyperplasia of the lymphoid follicles and obstruction of the appendiceal lumen [3]. 

 The incidence of *Salmonella* infection in renal transplant recipients ranges from 0 to 5%, varying according to species that are endemic in the community [7]. Once the organism enters the alimentary tract and disseminates through blood or lymphatics, invasion into Peyer's patches and mesenteric lymph nodes results in any combination of inflammatory sites [8].

 However, immunosuppression with steroids will be expected to diminish lymphoid hyperplasia with obstruction of the appendiceal lumen less likely in the renal allograft recipient [9]. 

 This case is to our knowledge the first reported case of *Salmonella* appendicitis in a renal transplant recipient.

Delay in diagnosis of significant and serious intraabdominal pathology in immunosuppressed patients is believed to be related to masking of peritoneal irritation by the anti-inflammatory effects of steroids [10]. In contrast, our patient had clear evidence of peritonitis, and there was little difficulty in making a diagnosis of a surgical emergency.

 In conclusion, appendicitis remains rarely reported in renal transplant patients, and a high index of suspicion with appropriately timed surgical intervention will allow for successful treatment of this surgical emergency.
